# From bond to guardianship: a serial mediation model explaining how the human–pet bond protects against pet relinquishment

**DOI:** 10.3389/fvets.2026.1780360

**Published:** 2026-07-03

**Authors:** Huanhua Lu, Yawen Zhao, Zaina Jianaer, Xiaofei Xia

**Affiliations:** 1 School of Marxism, China University of Geosciences (Beijing), Beijing, China; 2 Department of Information Technology, Natural History Museum of China, Beijing, China

**Keywords:** human–pet bond, pet empathy, pet relinquishment, pet suffering perception, responsibility commitment, chain mediation analysis

## Abstract

**Introduction:**

Pet relinquishment is a major global animal welfare issue, yet the psychological mechanisms that protect against it remain insufficiently understood. Drawing on social bond theory and the cognitive–affective system framework, this study examined whether the human–pet bond influences attitudes toward pet relinquishment through a sequential cognitive–emotional pathway involving pet suffering perception and pet empathy.

**Methods:**

We first developed and validated a two-dimensional Pet Relinquishment Attitude Scale (PRAS), comprising relinquishment rationalization and responsibility commitment, tailored for collectivistic contexts. Survey data were collected from 444 pet owners. Correlation analyses and serial mediation analyses were conducted to test the proposed model.

**Results:**

A stronger human–pet bond was significantly associated with higher responsibility commitment (*r* = 0.541, *p* < 0.001), but was not significantly associated with relinquishment rationalization (*r* = −0.081, *p* = 0.087). Serial mediation analysis revealed a significant indirect pathway from human–pet bond to responsibility commitment through pet suffering perception and pet empathy (b = 0.031, SE = 0.009, 95% CI [0.014, 0.049]). Specifically, a stronger human–pet bond increased owners’ sensitivity to their pets’ distress, which in turn evoked greater pet empathy and strengthened responsibility commitment.

**Discussion:**

The findings suggest that the protective role of the human–pet bond operates through a cognitive–emotional mechanism involving both pet suffering perception and pet empathy. By identifying this pathway and introducing the PRAS as a culturally relevant measure of pet relinquishment attitudes, the study contributes to the literature on responsible pet ownership and provides practical implications for pet relinquishment prevention.

## Introduction

Pet relinquishment has become an increasingly prominent global issue affecting both animal welfare and social management systems (1–5). Each year, millions of companion animals are surrendered to shelters or abandoned by their owners. In the United States alone, approximately 5.8 million companion animals enter shelters annually, including about 2.9 million dogs and 2.9 million cats (1). Similar patterns have been documented in Europe and other regions, where animal shelters face persistent capacity pressures due to owner relinquishment and stray animal intake (2–5). Such relinquishment not only exposes animals to significant stress and welfare risks but also places substantial burdens on shelter systems and public management resources (6).

Understanding the psychological factors underlying relinquishment decisions is therefore essential for developing effective prevention strategies. Attitudes toward pet relinquishment refer to relatively stable evaluations and behavioral tendencies regarding the act of giving up the care of companion animals (7, 8). According to the Theory of Planned Behavior, attitudes toward a behavior represent a proximal determinant of behavioral intentions and subsequent behavior (9). Examining how such attitudes are formed may therefore provide an important perspective for predicting and preventing relinquishment behavior.

Previous research has identified numerous external risk factors associated with pet relinquishment, including financial stress, housing restrictions, veterinary costs, and behavioral problems in pets (2, 10–12). More recent studies have further highlighted the role of socioeconomic vulnerability and service availability in shaping relinquishment patterns across communities (13, 14, 58). However, these external factors cannot fully explain why owners facing similar circumstances make very different decisions—some persist in caring for their pets, whereas others relinquish them. This divergence suggests that internal psychological processes may play an important role in shaping attitudes toward relinquishment.

One potential protective factor is the human–pet bond, defined as the dynamic and mutually beneficial relationship that develops between people and their companion animals through ongoing interactions (15). Strong human–pet bonds have been associated with increased caregiving behaviors, greater investment in animal welfare, and stronger perceptions of pets as family members (8, 16–18). Recent research further emphasizes that human–animal relationships may generate reciprocal psychological benefits for both humans and animals, including emotional regulation, companionship, and social support (19, 20).

However, despite the recognized importance of human–pet relationships, relatively little research has examined how the human–pet bond may influence attitudes toward pet relinquishment and the psychological mechanisms through which this influence occurs. The present study aims to address this gap by examining whether and how the human–pet bond shapes individuals’ attitudes toward pet relinquishment. Specifically, we propose that the human–pet bond influences relinquishment attitudes through a sequential psychological process involving cognitive and emotional mechanisms.

### Human–pet bond and attitudes toward pet relinquishment

Human–pet bond refers to a stable relationship encompassing emotional, cognitive, and behavioral dimensions that develops through long-term interactions between humans and their companion animals (15). This bond reflects not only emotional attachment but also patterns of mutual responsiveness and caregiving that shape everyday interactions between owners and their pets. A high-quality human–pet bond is typically characterized by strong emotional attachment, cognitive connectedness, and behavioral interdependence, with many owners perceiving their pets as family members and demonstrating substantial investment in their welfare (21–23, 53). Recent studies further emphasize that companion animals often fulfill important social and psychological roles, including providing emotional support, companionship, and a sense of relational continuity in daily life (17, 19, 20, 24, 25, 57).

From a theoretical perspective, social-bond theory posits that strong relational ties generate commitment and normative constraints that discourage behaviors harmful to the relationship (26, 27). When applied to human–pet relationships, this framework suggests that individuals who develop strong affective bonds with their pets may experience a heightened sense of responsibility and moral obligation toward them. Such relational commitment can shape caregiving motivations and influence decisions regarding the pet’s welfare. Empirical research provides evidence consistent with this perspective. Stronger human–pet bonds have been associated with higher levels of caregiving behavior, including greater willingness to invest in veterinary care, health monitoring, and daily welfare-related activities (8, 16, 18). Research in neuroscience also suggests that close interaction with companion animals may activate neurobiological systems associated with attachment and reward, thereby reinforcing caregiving motivation and emotional commitment (28). These findings indicate that individuals who form stronger emotional bonds with their pets are generally more motivated to maintain and protect the relationship, even when caregiving challenges arise.

Moreover, the strength and nature of the human–pet bond may vary across different companion animal species and ownership contexts. Previous research suggests that animals with higher levels of social interaction—such as dogs—often elicit stronger attachment responses from their owners compared with less interactive species (15, 22). In addition, households that own multiple pets may exhibit differentiated bonding patterns, with some animals receiving greater emotional investment than others (22, 23, 29). Although such variation highlights the complexity of human–pet relationships, the central role of emotional bonding in motivating caregiving behavior appears consistent across different ownership contexts. Therefore, the overall strength of the human–pet bond is expected to play a key role in shaping individuals’ attitudes toward relinquishment.

Based on this reasoning, individuals who develop stronger emotional bonds with their companion animals may perceive relinquishment as more inconsistent with their relational commitments and moral responsibilities. Consequently, they are likely to show lower acceptance of pet relinquishment behavior.

*Hypothesis 1 (H1)*: Owners with stronger human–pet bonds are less likely to accept pet relinquishment.

### The mediating role of pet suffering perception

Pet suffering perception involves an owner’s ability to detect and interpret signs of a companion animal’s physical or emotional distress (30). Within human–animal interactions, this perceptual sensitivity forms the cognitive foundation for subsequent emotional and attitudinal responses. Attention allocation theory suggests that cognitive resources are limited and preferentially directed toward stimuli that are perceived as significant or valued (31). Accordingly, owners with positive human–pet bonds—due to their emotional investment—are likely to prioritize their pets’ welfare, showing heightened sensitivity to subtle behavioral changes and signs of distress. Empirical studies indicate that owners with stronger attachment are indeed more accurate or attentive in identifying the stress signals and discomfort manifestations of their dogs (32, 33).

Furthermore, the cognitive–affective system theory posits that individuals’ cognitive appraisals of situations directly influence their attitude formation (34). Kielland et al. (30) found that enhancing awareness of animal suffering significantly improved people’s attitudes toward animal welfare. Recent research also indicates that people’s perception of animals’ cognitive abilities and capacity for suffering is closely linked to stronger emotional attachment and caregiving concern toward companion animals (35). In the context of pet relinquishment decision-making, heightened perception of pet suffering will make the owner more deeply aware of the possible painful consequences that relinquishment may bring to the pet, thereby strengthening their attitude against relinquishment. Thus, we propose the following hypothesis:

*Hypothesis 2 (H2)*: Pet suffering perception will mediate the relationship between human–pet bond and attitudes toward pet relinquishment, such that stronger human–pet bonds will be associated with greater perception of pet suffering, which in turn will be associated with lower acceptance of pet relinquishment.

### The mediating role of pet empathy

Pet empathy encompasses understanding an animal’s emotional state and sharing its affective experience, including both cognitive perspective-taking and affective resonance components (36). Previous studies have revealed that close relationships between individuals and others can increase perceived self–other merging, thereby strengthening the response of concern and assistance to others (37, 38). In human–pet interactions, a deep emotional bond facilitates owners’ ability to adopt their pet’s perspective, thereby increasing empathic responsiveness. Research has shown that owners with stronger emotional attachment to pets report higher levels of animal-directed empathy (39).

Moreover, the empathy–altruism hypothesis proposes that empathic concern elicits altruistic motivation, promoting prosocial and caregiving behaviors (Batson, 1991). Previous studies have confirmed that empathy is a key emotional mechanism underlying pro-animal attitudes and caregiving commitment (19, 24, 36, 40–43) (Telle and Pfister, 2014). For example, Taylor and Signal (43) found that individuals with higher empathy traits held more positive attitudes toward animal welfare. In relinquishment contexts, strong pet empathy enables owners to vividly imagine the fear, confusion, and pain their pets might experience after being abandoned. This emotional simulation evokes moral discomfort and guilt, strengthening resistance to pet relinquishment. Thus, we propose the following hypothesis:

*Hypothesis 3 (H3)*: Pet empathy will mediate the relationship between human–pet bond and attitudes toward pet relinquishment, such that stronger human–pet bonds will be associated with higher levels of pet empathy, which in turn will be associated with lower acceptance of pet relinquishment.

### An integrated sequential mediation model

Based on the cognitive–affective system theory, psychological processes often follow a sequential pattern of cognitive appraisal → emotional response → attitude formation (34, 44). This model highlights that cognitive evaluation of another’s suffering is a necessary precondition for eliciting empathic emotion. Studies have indicated that recognition of animal suffering can stimulate empathic concern, which in turn elevates moral concern and action on behalf of animals (19, 45), providing evidence for a chained relationship between suffering perception and empathy. In human–pet interactions, this mechanism may manifest as a full sequential mediation: a close human–pet bond enhances cognitive sensitivity to pet suffering (pet suffering perception), which then evokes emotional responses (pet empathy), ultimately translating into a stronger caregiving commitment and reduced acceptance of relinquishment. This “relationship → perception → emotion → attitude” pathway provides a systematic account of how the human–pet bond shapes attitudes toward relinquishment. Thus, we propose the following hypothesis:

*Hypothesis 4 (H4)*: Pet suffering perception and pet empathy will form a sequential mediation pathway linking human–pet bond and attitudes toward pet relinquishment, such that stronger human–pet bonds will increase pet suffering perception, which will in turn enhance pet empathy and ultimately reduce acceptance of pet relinquishment.

### The present study

**Figure 1:**
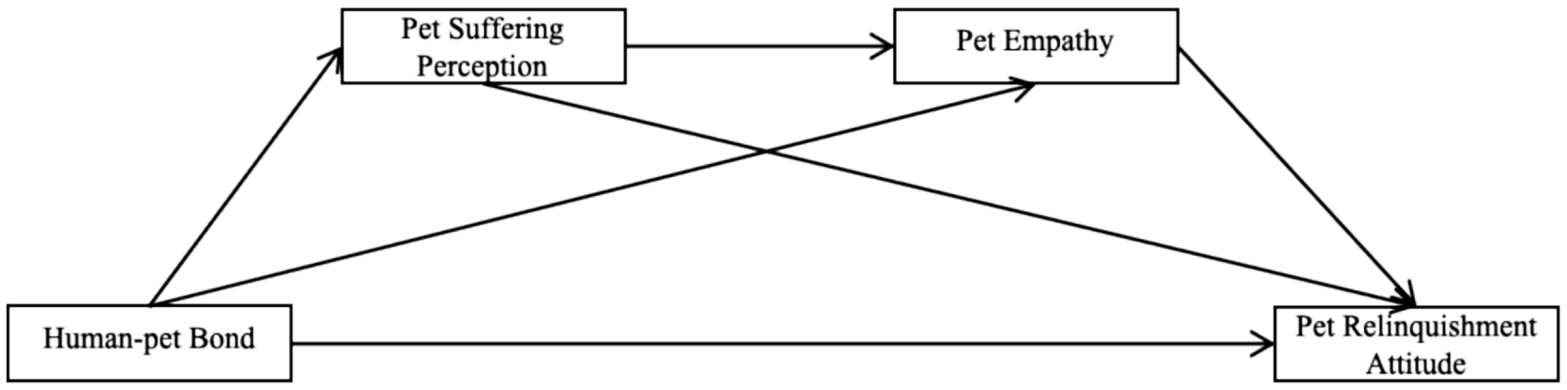
Diagram of hypothesized relationships between variables.

The present study aims to systematically examine whether and how the human–pet bond influences individuals’ attitudes toward pet relinquishment. Specifically, we propose a sequential mediation model (Figure 1) in which a positive human–pet bond enhances cognitive sensitivity to pet suffering, which subsequently evokes greater empathic concern for the pet, ultimately reinforcing opposition to relinquishment. To test this theoretical model, the study developed and validated a Pet Relinquishment Attitude Scale (PRAS) suitable for use in collectivistic Eastern cultural contexts; then, a questionnaire survey was conducted to test the proposed sequential mediation model. By revealing the internal psychological mechanisms underlying the link between human–pet bonds and pet relinquishment attitudes, this study provides new theoretical insights into psychological protective factors that prevent pet relinquishment and offers empirical support for developing interventions to promote responsible pet ownership and animal welfare.

## Pilot study—development and validation of the pet relinquishment attitude scale

### The necessity of developing a new pet relinquishment attitude scale

Existing studies generally conceptualize attitudes toward pet relinquishment as an individual’s overall evaluative tendency toward giving up a pet, encompassing cognitive, affective, and moral dimensions (7). The level of this attitude not only reflects the degree to which one justifies the act of relinquishment but also indicates one’s recognition of the moral responsibility and sense of obligation in pet care (7). Among the existing measures, the Attitudes Toward Pet Relinquishment (ATPR) scale developed by Jacobetty et al. (7) is one of the most representative tools. The scale includes two dimensions—pragmatism and lack of obligation—which conceptualize relinquishment attitudes primarily from the perspectives of behavioral rationalization and personal responsibility deficiency.

The “pragmatism” dimension in the ATPR scale (7) reflects the general acceptance of pet relinquishment under practical circumstances, but the original ATPR items are relatively broad and abstract in describing such situations (e.g., when family situations make continued care difficult). In the present study, we aimed to develop items that more concretely capture specific situational contexts in which relinquishment may be perceived as understandable, such as expensive veterinary treatment, housing restrictions, severe behavioral problems, or owner allergies. Because these items capture individuals’ tendency to cognitively justify relinquishment under particular circumstances, the dimension was labeled “relinquishment rationalization” rather than “pragmatism.”

Moreover, the ATPR scale (7) was developed in a Western individualistic cultural context, where the notion of “obligation” emphasizes personal responsibility. This perspective may not fully capture the relational and affective obligations emphasized in collectivistic cultures, where pets are often viewed as members of an emotional family community (23, 46). Directly applying “lack of obligation” dimension risks overlooking the unique cultural connotations of pet relinquishment attitudes in collectivistic societies.

Besides, from the perspective of the present study’s theoretical framework, the ATPR scale does not align well with our research objectives. The current study aims to test a sequential mediation model in which the human–pet bond influences pet suffering perception and pet empathy, which in turn affect attitudes toward pet relinquishment. In this model, empathy is theorized to foster a positive sense of responsibility toward the pet. However, the “lack of obligation” dimension of the ATPR scale measures a deficit in responsibility rather than the presence of moral commitment, making it less suitable for capturing the type of prosocial, emotionally grounded responsibility central to our framework.

Therefore, it is necessary to develop a new PRAS that better reflects the collectivistic cultural context and fits the conceptual model of this study.

### Methods

#### Initial construction of scale dimensions

To construct a theoretically grounded and culturally relevant scale, we combined literature analysis and qualitative interviews to identify and refine potential dimensions of pet relinquishment attitudes.

First, literature review clarified the core conceptual components of relinquishment attitudes. Western studies [e.g., (7)] have identified “pragmatism” and “lack of obligation” as two fundamental dimensions, providing an important theoretical starting point for the present research. Second, to address the cultural limitations of Western measures and ensure that the dimensions and items accurately reflect pet owners’ psychological perspectives in collectivistic cultures, we conducted semi-structured interviews with 12 participants, including 8 pet owners, 2 pet store operators, and 2 animal welfare volunteers. The interviews focused on questions such as: “Under what circumstances do you think giving up a pet is understandable?” and “How do you view the responsibilities associated with pet ownership?”

All interviews were audio-recorded and transcribed verbatim. The qualitative data were analyzed using a thematic analysis approach. Two researchers independently reviewed the transcripts, conducted open coding to identify meaningful statements related to attitudes toward pet relinquishment, and grouped similar codes into preliminary categories. Through iterative comparison and discussion, these categories were refined into broader themes reflecting recurring patterns in participants’ reasoning and emotional responses. Discrepancies between coders were resolved through discussion until consensus was reached.

Analysis of the interview data revealed two distinct cognitive–emotional tendencies: (1) relinquishment rationalization — participants often weighed the reasonableness or acceptability of relinquishment in response to specific challenges (e.g., high veterinary costs, housing restrictions, or serious behavioral problems in pets); (2) responsibility commitment — participants expressed a strong belief that pets are family members and should not be abandoned under any circumstances.

Integrating the findings from both literature and interviews, we established two core dimensions of the PRAS: (1) relinquishment rationalization: refers to the extent to which individuals cognitively evaluate relinquishment as reasonable, acceptable, or understandable when facing internal or external difficulties. (2) responsibility commitment: refers to the degree to which individuals identify with the long-term, unconditional emotional and moral obligations of pet care. This dimension emphasizes the internalization of the pet as a “family member,” along with the proactive caregiving and enduring moral commitment that arise from this identification.

#### Item development and content validity evaluation

*Item development*. Based on the two theoretical dimensions above, we generated an initial pool of items from two main sources: (1) transformation of typical situations and viewpoints derived from qualitative interviews; (2) adaptation and cultural localization of commonly reported reasons for pet relinquishment identified in prior domestic and international studies [e.g., (2, 6, 10–12)].

We also reviewed items from the ATPR (7) to ensure conceptual alignment with existing measures. However, rather than directly adopting ATPR items (7), we developed new items tailored to the present study’s conceptual framework and cultural context. Specifically, items under the relinquishment rationalization dimension focused on concrete situational justifications for relinquishment, while items under the responsibility commitment dimension emphasized the moral belief that pets should be treated as family members and cared for unconditionally (54).

The initial item pool consisted of 12 items, with 6 items for relinquishment rationalization and 6 items for responsibility commitment. All items were rated on a five-point Likert scale ranging from 1 (“strongly disagree”) to 5 (“strongly agree”).

*Expert content validity evaluation*. To assess the content validity of the preliminary scale, a panel of five experts was invited to review the items, including one animal behavior researcher, two psychology professors, and two senior practitioners in animal welfare. Experts evaluated each item in terms of its relevance to the intended dimension, as well as its clarity and precision, and provided suggestions for improvement. Based on their suggestions, minor wording adjustments were made to improve clarity and remove potential ambiguities, and two items deemed unsuitable were deleted. The resulting initial version of the PRAS consisted of 10 items, with five items under relinquishment rationalization and five items under responsibility commitment. The final items and their factor loadings are presented in the Results section together with the exploratory factor analysis (EFA).

#### Participants and procedure for psychometric validation

The validation process was conducted using two independent samples. Sample 1 consisted of 114 participants and was employed for the EFA. This sample size met the recommended participant-to-item ratio of at least 10:1, ensuring adequacy for factor exploration. Sample 2 comprised 444 participants, the same group as in the main study, and was used for the confirmatory factor analysis (CFA) as well as criterion validity testing. Employing this independent sample for CFA provided a robust test of the factor structure, while also enabling hypothesis testing in the main study.

Both samples were recruited using exactly the same procedure. A non-probability sampling strategy that combined convenience and snowball approaches was employed. We first posted recruitment messages on social media platforms (Weibo, Xiaohongshu, Douyin) and in offline pet-related venues (e.g., pet cafe, pet hospital) to conveniently enroll pet-owning young adults. We then offered a referral incentive—each participant who successfully recruited one eligible peer received a small pet gift for both parties—thereby generating successive waves of referrals and snowballing the sample. The demographic characteristics of participants in Sample 1 and Sample 2 are presented in Tables 1, 2 respectively. The study was approved by China University of Geosciences (Beijing) Psychological Ethics Committee (No. 20250705). All participants provided written informed consent before participating in this study.

**Table 1:** Participant demographics of sample 1 (*n* = 114).

Characteristic	Subcategory	Value
Gender	Male	50 (43.86%)
	Female	64 (56.14%)
Age	M	19.17
	SD	1.21
Place of residence	Urban	16 (14.04%)
	Rural	98 (85.96%)
Educational attainment	High school or below	15 (13.16%)
	College degree or above:	99 (86.84%)
Family socioeconomic status	Low	40 (35.09%)
	Middle	64 (56.14%)
	High	10 (8.77%)

Family socioeconomic status(SES) was measured on a 10-point scale. Participants were categorized into three groups based on their scores: Low SES (scores 1–3), Middle SES (scores 4–7), and High SES (scores 8–10).

**Table 2:** Participant demographics of sample 2 (*n* = 444).

Characteristic	Subcategory	Value
Gender	Male	273 (61.49%)
	Female	171 (38.51%)
Age	M	20.36
	SD	4.56
Place of residence	Urban	248 (55.86%)
	Rural	196 (44.14%)
Educational attainment	High school or below	36 (8.11%)
	College degree or above	408 (91.89%)
Family socioeconomic status	Low	115 (25.90%)
	Middle	306 (68.92%)
	High	23 (5.18%)
Primary type of pet owned	Cat	85 (19.14%)
	Dog	162 (36.49%)
	Other (e.g., fish, birds, reptiles)	197 (44.37%)
Ever relinquished a pet	Yes	63 (14.19%)
	No	381 (85.81%)

Family socioeconomic status (SES) was measured on a 10-point scale. Participants were categorized into three groups based on their scores: Low SES (scores 1–3), Middle SES (scores 4–7), and High SES (scores 8–10). Participants were asked to indicate their primary type of pet using a single-choice item (cat, dog, or other). Because only one option could be selected, percentages sum to 100%.

All participants completed the 10-item preliminary scale. To assess criterion validity, participants in Sample 2 also completed the ATPR scale (7), which measures attitudes toward pet relinquishment across two distinct dimensions: lack of obligation and pragmatism. The lack of obligation dimension (4 items) reflects the belief that owners are not necessarily duty-bound to retain a pet, whereas the pragmatism dimension (3 items) reflects practical or utilitarian considerations that frame relinquishment as a viable option under challenging circumstances. All seven items are rated on a 5-point Likert scale. In the present study, Cronbach’s *α* was 0.703 for the pragmatism dimension and was 0.947 for the lack of obligation dimension.

#### Statistical analysis

To examine the psychometric properties of the scale, a comprehensive statistical analysis was conducted. First, an EFA using principal component analysis with Kaiser-normalized Oblimin rotation was performed on Sample 1 to explore the underlying factor structure (55). Subsequently, a CFA was applied to Sample 2 to verify the two-factor model identified in the EFA. The internal consistency was assessed using Cronbach’s alpha in both Sample 1 and Sample 2. Furthermore, criterion validity was evaluated by calculating Pearson correlation coefficients between the PRAS and the ATPR scale.

### Results

#### Exploratory factor analysis (EFA) and initial reliability

An EFA was conducted on Sample 1. The Kaiser–Meyer–Olkin measure indicated adequate sampling adequacy (KMO = 0.845), and Bartlett’s test of sphericity was significant (χ^2^(45) = 737.797, *p* < 0.001), supporting the suitability of the data for factor analysis. The analysis yielded a two-factor solution that explained 71.495% of the total variance. As shown in Table 3, items loaded strongly on their intended factors. Factor 1, labeled *relinquishment rationalization*, consisted of five items (e.g., “Relinquishing a pet is understandable if it has a severe and costly-to-treat illness”), with primary loadings ranging from 0.551 to 0.911. Factor 2, labeled *responsibility commitment*, also consisted of five items (e.g., “Pets are family members and should never be relinquished under any circumstances”), with primary loadings ranging from 0.735 to 0.870. Although several items showed secondary loadings on the other factor, these loadings were smaller in magnitude and followed the expected theoretical direction, supporting the conceptual distinction between the two constructs. The two factors were moderately and negatively correlated (*r* = −0.316), indicating that although the constructs are related, they represent distinguishable dimensions of attitudes toward pet relinquishment.

**Table 3:** Exploratory factor analysis results (pattern matrix).

Item	Factor A: relinquishment rationalization	Factor B: responsibility commitment
A1. Relinquishing a pet is understandable if it has a severe and costly-to-treat illness.	**0.911**	0.123
A2. Relinquishment is justifiable when moving to a new house that does not allow pets.	**0.899**	0.159
A3. Relinquishment is an option for pets with severe and uncorrectable behavioral issues (e.g., aggression, excessive barking).	**0.767**	−0.193
A4. Relinquishment is reasonable due to owner allergies or pregnancy.	**0.815**	−0.098
A5. Sending a pet to a shelter does not constitute true “relinquishment.”	**0.551**	−0.423
B1. Pets are family members and should never be relinquished under any circumstances.	0.044	**0.821**
B2. All potential difficulties and costs of pet ownership should be considered beforehand.	−0.256	**0.735**
B3. The primary responsibility for addressing a pet’s behavioral issues lies with the owner, not the pet itself.	−0.052	**0.868**
B4. Casually relinquishing a pet is an irresponsible and unethical act.	0.067	**0.870**
B5. Governments should impose stricter penalties for irresponsible pet relinquishment.	0.083	**0.843**

Extraction method: Principal Component Analysis. Rotation method: Kaiser-normalized Oblimin rotation. Factor correlation matrix showed a negative correlation between the two factors (*r* = −0.316). Bolded loadings indicate the primary factor on which each item was assigned.

Internal consistency analysis conducted on this initial sample indicated that both dimensions of the scale demonstrated good reliability at this early stage: relinquishment rationalization (*α* = 0.880) and responsibility commitment (α = 0.894).

#### Confirmatory factor analysis (CFA) and cross-sample reliability

The CFA performed on the independent Sample 2 tested the two-factor model identified by the EFA. The model demonstrated acceptable fit to the data: χ^2^/df = 4.895, CFI = 0.915, TLI = 0.872, RMSEA = 0.094 (90% CI [0.079, 0.109]), SRMR = 0.083 (56).

To further evaluate the convergent validity of the scale, composite reliability (CR) and average variance extracted (AVE) were calculated for each latent factor. The results indicated satisfactory values for both dimensions (relinquishment rationalization: CR = 0.844, AVE = 0.531; responsibility commitment: CR = 0.865, AVE = 0.566), exceeding the recommended thresholds of CR > 0.70 and AVE > 0.50, suggesting adequate convergent validity.

Discriminant validity was examined using the Fornell–Larcker criterion. The square roots of the AVE values for each factor (relinquishment rationalization: 0.729; responsibility commitment: 0.752) were greater than the correlation between the two latent constructs (*r* = −0.153), indicating that relinquishment rationalization and responsibility commitment represent empirically distinct dimensions.

In this validation sample, the internal consistency reliabilities for both dimensions of the scale remained excellent: the relinquishment rationalization dimension (α = 0.840) and the responsibility commitment dimension (α = 0.843).

These results jointly support the reliability and construct validity of the two-factor structure of the PRAS.

#### Criterion validity

The relinquishment rationalization dimension was positively correlated with the pragmatism dimension of the ATPR scale (*r* = 0.375, *p* < 0.001), and the responsibility commitment dimension was negatively correlated with the lack of obligation dimension of the ATPR scale (*r* = −0.126, *p* = 0.008), which provided strong evidence for good criterion validity.

#### Summary of the pilot study

The pilot study successfully developed and validated a psychometrically sound, two-dimensional instrument: the PRAS. The rigorous process, spanning qualitative item generation, expert review, and quantitative validation across two independent samples, ensures the scale’s content validity, construct validity, criterion validity and reliability (60). This validated two-dimensional scale allows for a comprehensive and nuanced investigation of the drivers of pet relinquishment attitude in the main study.

## Main study: the association of human-pet bond, perceived pet suffering, pet empathy, and pet relinquishment attitude

### Methods

#### Participants

The main study used the sample 2 from the pilot study as the participants. The recruitment procedure for participants has been described in detail in the pilot study. Data were collected via the Wenjuanxing online survey platform, yielding 463 responses. After excluding invalid responses (e.g., patterned answering, abnormally short completion times), 444 valid samples remained. Among them, 273 were male and 171 were female. The mean age was 20.36 years (SD = 4.56). The study was approved by China University of Geosciences (Beijing) Psychological Ethics Committee (No. 20250705). All participants provided written informed consent before participating in this study. Participants were required to have current or previous experience of pet ownership. Participants were not instructed to complete the questionnaire with reference to a specific individual pet. Instead, they were asked to respond based on their general experiences and relationships with pets they currently own or had owned in the past.

#### Measures

*Human–Pet Bond*. Human–pet bond was operationalized using the Brief Lexington Attachment to Pets Scale (B-LAPS) (47). This scale is a revised version of the original Lexington Attachment to Pets Scale (LAPS) developed by Johnson et al. (21). The B-LAPS consists of 11 items forming a unidimensional structure, which contrasts with the original 23-item instrument comprising three dimensions (21, 47). Responses are recorded on a five-point Likert scale. Participants completed the scale based on their general experiences and relationships with pets rather than referring to a specific individual pet. The total score, derived from summing all item responses, provides a global measure of owner-pet relationship closeness, with higher scores indicating a stronger level of attachment. Cronbach’s *α* was 0.956. In present study, LAPS scores were used as the observed indicator of the human–pet bond.

*Pet Suffering Perception*. The Pet Suffering Perception Scale was developed by modifying items related to animal suffering perception from the Animal Attitude Scale (48), and it is used to assess an individual’s perception of suffering in pets. The scale comprises three items [e.g., “I find it easy to perceive and identify when my pet shows signs of physical discomfort (such as decreased appetite, limping, or rapid/irregular breathing)”]. Each item is rated on a 5-point Likert scale. The scores of all items were added together as the total score of the scale. A higher total score indicates a greater ability of the individual to perceive suffering in pets. The Cronbach’s *α* was 0.913.

*Pet Empathy*. The Pet Empathy Scale including three items was designed to assess an individual’s feelings of concern, sympathy, and compassion towards pets (e.g., “I feel extremely worried when my pet is ill or injured”). This scale was adapted from the short version of the Empathic Concern Index (49, 50) and used a 5-point scoring system. The scores of all items were added together as the total score of the scale. Higher total scores indicate a greater tendency for the individual to experience empathy towards pets. The Cronbach’s α was 0.946.

*Pet Relinquishment Attitude*. The PRAS was used to assess attitudes toward pet relinquishment. The scale comprises two dimensions: each dimension consists of 5 items rated on a 5-point Likert scale. The relinquishment rationalization dimension was measured using five items describing situations in which external constraints may justify pet relinquishment, including high veterinary costs due to serious illness, housing restrictions after moving, severe uncorrectable behavioral problems, and owner health-related issues such as allergies or pregnancy. In the present study, “relinquishment rationalization” refers to individuals’ cognitive evaluations of the acceptability or justifiability of relinquishment under specific circumstances, rather than a defensive psychological mechanism in the psychoanalytic sense. The higher the score in the relinquishment rationalization dimension, the more an individual believes that relinquishment is reasonable under stressful circumstances; the higher the score in the responsibility commitment dimension, the more the individual believes that pets are one of the family members and that keeping them is a responsibility. In this study, Cronbach’s α was 0.843 for the responsibility commitment dimension and 0.840 for the relinquishment rationalization dimension.

*Demographic Information*. Participants reported demographic information including gender, age, educational attainment, family socioeconomic status, and place of residence. Participants were also asked to report their primary type of pet and whether they had any experience of relinquishing pets. All the above information was presented in Table 2. In the chain mediation analysis, all the above information was used as control variables.

#### Statistical analysis

First, descriptive statistics and correlation analyses were conducted using SPSS 27.0. Subsequently, we performed a chain mediation analysis to evaluate the hypothesized theoretical model (Figure 1) (59). The significance of mediation effects was tested using a bootstrap approach with 5,000 resamples.

### Results

#### Common method bias test

To examine the potential influence of common method variance, Harman’s single-factor test was conducted. All measurement items from the four main constructs included in the study—LAPS, pet suffering perception, pet empathy, and PRAS—were entered into the analysis. The results indicated that the first factor accounted for 35.22% of the variance, below the 40% threshold (51), suggesting no serious common method bias.

#### Correlations analysis

As presented in Table 4, correlation analysis revealed that LAPS scores which were used as the observed indicator of the human–pet bond were significantly positively correlated with responsibility commitment (*r* = 0.541, *p* < 0.001), but not significantly correlated with relinquishment rationalization dimension (*r* = −0.081, *p* = 0.087). The Steiger’s Z test for two correlation coefficients showed that the correlation between LAPS scores and responsibility commitment (*r* = 0.541, *p* < 0.001) was significantly stronger than its correlation with relinquishment rationalization (*r* = −0.081, *p* = 0.087), with the difference being statistically significant (Z = 9.42, *p* < 0.001).

**Table 4:** Correlations among main variables.

Variables	1	2	3	4	5
1. LAPS scores	—				
2. Perceived pet suffering	0.498***	—			
3. Pet empathy	0.560***	0.757***	—		
4. Relinquishment rationalization	−0.081	−0.120*	−0.152**	—	
5. Responsibility commitment	0.541***	0.632***	0.663***	−0.148**	—

1 = LAPS scores, 2 = Perceived Pet Suffering, 3 = Pet Empathy, 4 = Relinquishment Rationalization, 5 = Responsibility Commitment, LAPS scores represent the human–pet bond; **p < 0.01, ***p < 0.001.

Both pet suffering perception (*r* = 0.632, *p* < 0.001) and pet empathy (*r* = 0.663, *p* < 0.001) are significantly positively correlated with responsibility commitment, but significantly negatively correlated with relinquishment rationalization dimension (pet suffering perception: *r* = −0.120, *p* = 0.011; pet empathy: *r* = −0.152, *p* = 0.001).

Additionally, the LAPS scores were significantly positively correlated with pet suffering perception (*r* = 0.498, *p* < 0.001) and pet empathy (*r* = 0.560, *p* < 0.001). Pet suffering perception was also significantly positively correlated with pet empathy (*r* = 0.757, *p* < 0.001). Relinquishment rationalization was significantly negatively correlated with responsibility commitment (*r* = −0.148, *p* = 0.002).

Because LAPS scores were not significantly correlated with the relinquishment rationalization dimension in the present sample, subsequent mediation analyses focused on the responsibility commitment dimension, which reflects the owner’s perceived moral obligation and commitment toward their pets.

#### Chain mediation analysis

A chain mediation analysis was conducted using PROCESS model 6, with LAPS scores which were used as the observed indicator of the human–pet bond as independent variable, pet suffering perception and pet empathy as mediators, responsibility commitment as the dependent variable, and participant’s gender, age, educational attainment, family socioeconomic status, place of residence, primary type of pet they owned and whether they had any experience of relinquishing pets as control variables.

**Figure 2:**
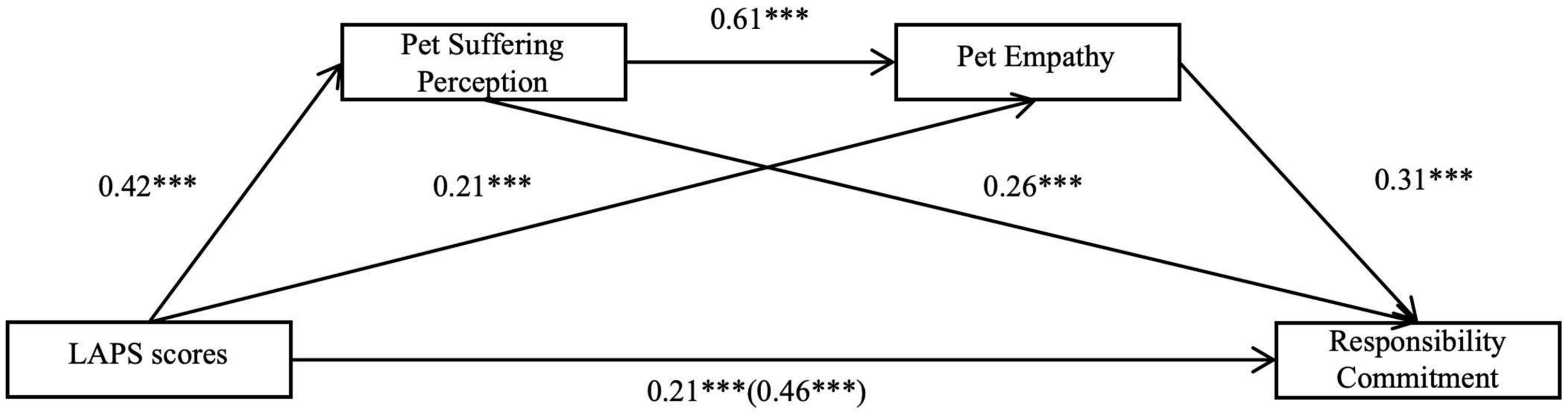
Path diagram of the chain mediation analysis. The values beside each arrow are the path coefficients of the chain mediation analysis. For the association between LAPS scores and responsibility commitment, the value in the parentheses is the total correlation, and the value outside the parentheses is the direct correlation after accounting for the mediators. LAPS scores were used as the operational indicator of human–pet bond. All values are standardized coefficients. **p < 0.05, ***p < 0.001.

The results showed that the total effect of the LAPS scores on responsibility commitment was statistically significant (b = 0.176, SE = 0.016, 95% CI [0.145, 0.207]). The direct effect was also significant (b = 0.079, SE = 0.016, 95% CI [0.048, 0.109]) when pet suffering perception and pet empathy were included as the first and second mediating variables (Table 5, Figure 2). The bootstrap simulation (*n* = 5,000) revealed that the LAPS scores had a significant chain mediation effect on the responsibility commitment through pet suffering perception and pet empathy (b = 0.031, SE = 0.009, 95% CI [0.014, 0.049]). Besides, there was a significant indirect effect via LAPS scores → pet suffering perception → responsibility commitment (b = 0.041, SE = 0.012, 95% CI [0.020, 0.067], Table 5), and a significant effect via LAPS scores → pet empathy → responsibility commitment (b = 0.026, SE = 0.009, 95% CI [0.011, 0.045]).

**Table 5:** Direct and indirect effects of human–pet bond on responsibility commitment.

Type of effect	Pathway	*b*	*SE*	95% CI	
				Lower	Upper
Total effect		0.176	0.016	0.145	0.207
Total indirect effect		0.097	0.016	0.067	0.131
Indirect effect	LAPS scores → PSP → RC	0.041	0.012	0.020	0.067
	LAPS scores → PE → RC	0.026	0.009	0.011	0.045
	LAPS scores → PSP → PE → RC	0.031	0.009	0.014	0.049
Direct Effect	LAPS scores → RC	0.079	0.016	0.048	0.109

LAPS scores represent the human–pet bond; PSP, Pet Suffering Perception; PE, Pet Empathy; RC, Responsibility Commitment; *b*, unstandardized regression coefficient; SE, standard error.

Overall, these findings highlight the importance of a dual-pathway mechanism in which close human–pet bond (as indexed by LAPS scores) enhance perception of pet suffering, which subsequently fosters pet empathy, ultimately, strengthens greater responsibility commitment toward pet.

To clarify the relationship between the empirical findings and the proposed hypotheses, we summarized the hypothesis testing outcomes based on the correlation and mediation analyses. First, human–pet bond (as indexed by LAPS scores) was significantly positively associated with the responsibility commitment dimension of pet relinquishment attitudes but was not significantly correlated with the relinquishment rationalization dimension in the present sample. These findings suggest that human–pet bonds (as indexed by LAPS scores) may be more strongly associated with responsibility commitment than with relinquishment rationalization. Therefore, H1 received partial support. Second, mediation analyses indicated that pet suffering perception significantly mediated the relationship between human–pet bond (as indexed by LAPS scores) and responsibility commitment, supporting H2. Third, pet empathy also significantly mediated the relationship between human–pet bond (as indexed by LAPS scores) and responsibility commitment, supporting H3. Finally, the sequential mediation pathway human–pet bond (as indexed by LAPS scores) → pet suffering perception → pet empathy → responsibility commitment was significant, supporting H4.

## Discussion

This study aimed to explore whether and how the human–pet bond influences attitudes toward pet relinquishment. The findings revealed a differentiated pattern: the human–pet bond was positively associated with the responsibility commitment dimension through a sequential mediation of pet suffering perception and pet empathy, whereas it was not significantly associated with the relinquishment rationalization dimension. These findings provide insight into the psychological pathways through which the human–pet bond may shape attitudes toward pet relinquishment.

One important contribution of this study is the finding that the human–pet bond was more strongly related to responsibility commitment than to relinquishment rationalization in the present data. Specifically, the results suggest that a stronger human–pet bond may foster an enduring emotional attachment and a sense of responsibility toward one’s pet. In contrast, the present findings did not provide evidence that the human–pet bond directly influences context-specific cost–benefit evaluations of relinquishment, such as those reflected in the relinquishment rationalization dimension. When owners form a close emotional bond with their pets, they may become more attentive to signs of discomfort or suffering experienced by the animal [i.e., enhanced pet suffering perception; (32, 33)]. This heightened awareness can subsequently evoke emotional resonance and compassion toward the pet [i.e., increased pet empathy; (19, 39)], which may then contribute to a stronger internalized sense of responsibility toward the pet’s well-being. This progression from emotional connection to cognitive awareness, emotional resonance, and ultimately moral commitment aligns with the sequential processing assumptions proposed by cognitive–affective system theory (34) and concretizes the abstract notion of responsibility commitment proposed in social connection theory (26, 52) into an observable psychological process.

At the same time, the human–pet bond was not significantly associated with the relinquishment rationalization dimension. The result suggests that the relationship between the human–pet bond and relinquishment rationalization may be weak or context-dependent. One possible explanation is that when individuals face substantial external constraints—such as financial difficulties or housing restrictions—decision-making may be influenced more strongly by situational pressures and practical considerations (10–12, 14). Another possible explanation concerns the conceptual distinction between emotional attachment and decision justification. Human–pet bond primarily reflects emotional closeness and relational commitment, whereas relinquishment rationalization captures cognitive evaluations regarding whether relinquishment may be acceptable under particular situational constraints. According to dual-process perspectives on decision-making, emotionally driven attachment processes and context-dependent utilitarian evaluations may operate relatively independently. Therefore, even owners who report strong emotional bonds with their pets may still regard relinquishment as understandable when facing severe financial burden, housing instability, health concerns, or caregiving limitations. Nevertheless, this finding does not imply that relinquishment rationalization is unimportant. Rather, it may reflect a distinct psychological process that is shaped by contextual factors beyond the emotional bond with the pet. From an applied perspective, these findings suggest that efforts to reduce pet relinquishment may benefit from addressing both psychological and contextual factors. On the one hand, interventions that strengthen the human–pet bond—such as encouraging positive human–animal interactions or promoting empathy toward pets—may help reinforce owners’ sense of responsibility toward their animals. On the other hand, interventions aimed at reducing external barriers to pet ownership, such as financial assistance for veterinary care or initiatives supporting pet-friendly housing, may help alleviate practical pressures that contribute to relinquishment (6, 13).

The findings may also provide insights into understanding cultural variations in pet relinquishment decisions. The responsibility commitment dimension—encompassing notions of familial belonging and relational responsibility—might precisely capture the core concept in collectivist cultures of viewing pets as members of an emotional community (11). The finding that the human–pet bond affects responsibility commitment through emotional pathways within Eastern cultural contexts suggests that fostering deep emotional bonds with pets to evoke empathy and thereby strengthen moral responsibility may be an especially effective strategy for reducing pet relinquishment.

### Theoretical contributions and practical implications

The present study contributes to the literature on human–animal relationships and pet relinquishment attitudes in three important ways. First, it developed and validated a culturally sensitive instrument designed to assess attitudes toward pet relinquishment within a collectivist cultural context. The two-dimensional structure of the scale distinguishes between responsibility commitment and relinquishment rationalization, providing a framework for examining different psychological processes underlying relinquishment attitudes. Second, the findings further extend previous research on the human–pet bond by suggesting that its influence on relinquishment attitudes may operate partly through increased sensitivity to pet suffering and enhanced empathy toward the animal. Third, the results indicate that the influence of the human–pet bond may be more strongly expressed through moral and emotional dimensions of pet ownership than in context-dependent rational evaluations of relinquishment.

From a practical perspective, these findings provide useful implications for organizations seeking to reduce pet relinquishment. Animal welfare organizations and shelters could consider educational programs that promote positive human–pet interactions and help owners recognize signs of distress in their animals. Initiatives that encourage empathy toward pets—such as narrative-based campaigns highlighting animals’ emotional experiences—may also strengthen owners’ sense of responsibility toward their pets. At the same time, practical support services may play an important role. For example, shelters and welfare organizations might provide information about pet-friendly housing options, offer behavioral training resources, or connect pet owners with financial assistance programs for veterinary care. Such efforts may help address both the psychological and practical factors that contribute to relinquishment.

More specifically, intervention programs may benefit from adopting tailored strategies based on owners’ underlying risk profiles. For owners showing lower emotional engagement with pets, interventions could focus on strengthening human–pet interaction and enhancing awareness of animals’ emotional and physical needs. For owners facing situational constraints (e.g., financial burden, housing instability, or caregiving difficulties), practical retention-oriented services—such as temporary fostering, veterinary support, or pet retention counseling—may be more effective than solely emphasizing moral responsibility. In addition, the PRAS developed in the present study may serve as a preliminary assessment tool to identify individuals with elevated relinquishment risk and facilitate earlier targeted support before actual relinquishment decisions occur.

### Limitations and future directions

Several limitations should be noted. First, the cross-sectional design limits causal inference; future research should employ longitudinal or experimental designs to test the proposed mediation pathway more rigorously. Second, the reliance on self-report measures may introduce social desirability bias, and future studies could incorporate behavioral measures or multi-source data to improve measurement validity. Third, the present study relied on voluntary participation, which may introduce self-selection bias. Individuals with stronger emotional bonds to their pets may have been more likely to participate, potentially influencing the observed relationships. Future studies could recruit participants through more diverse channels, such as veterinary clinics, animal shelters, or community pet services, in order to obtain a more representative sample. Fourth, although the questionnaire asked participants to report the species of pet they currently or previously owned, the present study did not examine whether differences between species influence the strength of the human–pet bond or attitudes toward pet relinquishment. Because emotional attachment to pets may vary across species, future research should explore potential species-specific differences in the psychological mechanisms underlying relinquishment attitudes. Fifth, while participants were asked whether they had ever relinquished a pet, the present study did not specifically recruit individuals with direct relinquishment experience. Including more participants who have previously relinquished pets—such as those recruited through veterinary clinics or animal shelters—may provide deeper insight into the psychological processes associated with relinquishment decisions. Sixth, this study did not collect detailed information about the timing of pet relinquishment experiences. The psychological processes involved in relinquishment may differ depending on whether relinquishment occurs shortly after acquisition (e.g., due to allergies or lifestyle mismatch) or after several years of ownership. Future research should examine how the duration of the human–pet relationship influences relinquishment attitudes. Finally, because the sample consisted primarily of Chinese young adults, the findings may not fully reflect factors that are more common among other demographic groups, such as older populations or individuals from different cultural contexts. Future studies including more diverse age groups and cross-cultural samples would help clarify the generalizability of the present findings and further validate the scale.

## Conclusion

This study identifies an underlying psychological pathway: a positive human–pet bond enhances individuals’ sensitivity to their pets’ suffering, which in turn fosters empathetic concern and ultimately strengthens owners’ sense of responsibility toward maintaining pet ownership. These findings deepen understanding of the psychological processes underlying pet relinquishment attitudes and may inform the development of more targeted intervention strategies that simultaneously strengthen emotional responsibility and reduce contextual barriers to pet retention.

## Data Availability

The original contributions presented in the study are publicly available. This data can be found here: https://osf.io/n49aq/overview?view_only=2e492f4a1a674d799bc1dcda7383e023.
