# Supplementary material for: From bond to guardianship: a serial mediation model explaining how the human–pet bond protects against pet relinquishment
Source: Front Vet Sci. 2026 Jul 3;13:1780360. doi: 10.3389/fvets.2026.1780360 (PMC13377691; doi:10.3389/fvets.2026.1780360)
Supplement: Supplementary file 1 [file Data_Sheet_1.DOCX]

**Introduction**: Please completed the scale based on your general experiences and relationships with pets you currently own or had owned in the past, rather than referring to a specific individual pet. **Please indicate your level of agreement with the following statements using a 5-point Likert scale (1 = Strongly Disagree, 2 = Disagree, 3 = Neutral, 4 = Agree, 5 = Strongly Agree).**

**Pet Relinquishment Attitude Scale**

1.Relinquishing a pet is understandable if it has a severe and costly-to-treat illness.

2.Relinquishment is justifiable when moving to a new house that does not allow pets.

3.Relinquishment is an option for pets with severe and uncorrectable behavioral issues (e.g., aggression, excessive barking).

4.Relinquishment is reasonable due to owner allergies or pregnancy.

5.Sending a pet to a shelter does not constitute true "relinquishment".

6.Pets are family members and should never be relinquished under any circumstances.

7.All potential difficulties and costs of pet ownership should be considered beforehand.

8.The primary responsibility for addressing a pet's behavioral issues lies with the owner, not the pet itself.

9.Casually relinquishing a pet is an irresponsible and unethical act.

10.Governments should impose stricter penalties for irresponsible pet relinquishment.

**The Brief Lexington Attachment to Pets Scale**

1.My pet means more to me than any of my friends.

2.I think my pet is just a pet.(rev)

3.My pet knows when I'm feeling bad.

4.I often talk to other people about my pet.

5.I believe that loving my pet helps me stay healthy.

6.Pets deserve as much respect as humans do.

7.My pet and I have a very close relationship.

8.I play with my pet quite often.

9.I consider my pet to be a great companion.

10.My pet makes me feel happy.

11.I consider my pet to be a friend.

**The Pet Empathy Scale**

1.I feel extremely worried when my pet is ill or injured

2. I feel deep sympathy for my pet when it is experiencing discomfort or distress.

3. Seeing my pet in a sad or uneasy state makes me feel compassion and a desire to comfort it.

**The Pet Suffering Perception Scale**

1. I can tell when my pet is feeling anxious or fearful based on its behavior (e.g., hiding, trembling, excessive vocalization).

2.I find it easy to perceive and identify when my pet shows signs of physical discomfort (such as decreased appetite, limping, or rapid/irregular breathing).

3. I can keenly perceive my pet's discomfort or pain.

**The Attitudes Toward Pet Relinquishment Scale (only used in pilot study)**

1.It’s irresponsible to keep a pet that doesn’t adjust to us.

2.There are family circumstances that force the pet’s relinquishment.

3.Sometimes there is nothing that can be done to keep the pet.

4.I would do anything to avoid relinquishing my animal.

5.Abandoning an animal is an irresponsible practice.

6.I would never abandon my pet.

7.Animals must be protected by law.

**Demographic Information**

1.Your gender?

A. Male

B. Female

2.Where are you from?

A. Urban area

B. Rural area

3.What is your highest level of education?

A. High school or below

B. college degree or above

4.Your age: ( ) years old

5.Please imagine a ladder representing the position of different families in your region/area:

The top of the ladder represents the families with the highest living standards in your region/area—those with the highest income, highest education level, and highest social status.

The bottom of the ladder represents the families with the lowest living standards in your region/area—those with the lowest income, lowest education level, and lowest social status.

Compared to other families in your region/area, please consider where you think your family currently stands on this ladder. Please fill in a number from 1 to 10, where 1 represents the lowest level and 10 represents the highest level.

Your rating: ( )


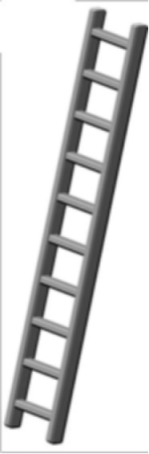


6.What primary type of pet do you currently own or have you owned in the past? (single-choice)

A. Cat

B. Dog

C. Other (e.g., fish, birds, reptiles)

7.Have you ever abandoned a pet?

A. Yes

B. No
